# Lesions caused by human and domestic and wild animal bites

**DOI:** 10.1590/0037-8682-0372-2022

**Published:** 2022-12-16

**Authors:** Vidal Haddad

**Affiliations:** 1 Universidade Estadual Paulista, Faculdade de Medicina de Botucatu, Botucatu, SP, Brasil.

**Keywords:** Animal bite, Bacterial infection, Fungal infection, Wild animal, Domestic animal, Human bite

## Abstract

Animal bites are a common problem in the emergency room. There are many reports of isolated cases (mainly of domestic mammals); however, texts with more comprehensive and general guidance on these kinds of bite injuries are necessary, including those caused by wild mammals, reptiles, and even fish. This review aims to update knowledge on this problem, which includes human and domestic and wild animal bites. Health teams in emergency care centers should be aware of the initial and late measures required to control this problem.

Animal bites in humans are potential sources of perforations, lacerations, and bacterial and fungal infections in later stages. Every bite introduces microorganisms into wounds, which can cause serious injuries to human skin, muscles, and even bones^1^ ^,^ ^2^ ^,^ ^3^ ^,^ ^4^ ^,^ ^5^.

These situations are rarely discussed in medical texts. There are many reports of isolated cases (mainly of domestic mammals); however, texts with more comprehensive and general guidance are needed on injuries caused by wild animals, including wild mammals, reptiles, and even fish^1^. The author discusses previously published cases of human and domestic and wild animal bites observed mainly in Ubatuba **C**ounty on the North Coast of the State of São Paulo, Brazil, and at the General Hospital of the Faculdade de Medicina de Botucatu, Universidade Estadual Paulista, located in the midwest region of São Paulo, in addition to some cases registered in other regions of the country.

One way to classify these injuries is to divide agents into wild and domestic animals and humans. Standardization of medical management is similar, with obvious exceptions, such as the prevention of rabies in mammalian bites^1^ ^,^ ^2^ ^,^ ^3^ ^,^ ^4^ ^,^ ^5^.

There are no reliable statistics regarding the number of animal bites in humans from around the world due to a lack of case notifications and domestic treatments being applied without victims seeking medical help, but it is known that animal bites are common in emergency rooms, particularly those involving domestic dogs^1^. In a study carried out in the USA, dogs were responsible for approximately 90% of animal bites, accounting for 1% of emergency room visits. In this series, human bites accounted for 2% of all animal bites^2^ ^,^ ^3^ ^,^ ^4^ ^,^ ^5^. Roaming domestic animals are common in urban areas, and wild animals often come into contact with human populations in municipalities with nature reserves and parks.

## DOMESTIC ANIMALS

**FIGURE 1:**
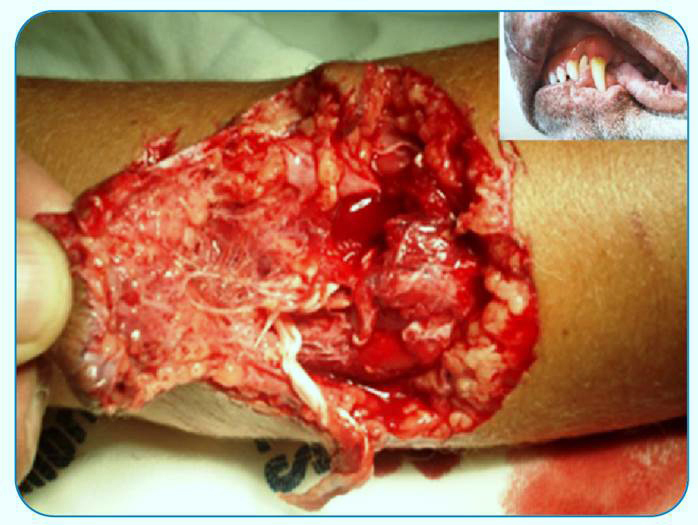
A dog bite that caused massive tissue destruction. In the detail: teething of a domestic dog.

Domestic dogs (*Canis lupus familiaris*) are the main cause of bites in humans^1^ ^-^ ^5^. Their teeth have a rhomboid shape, and although they can cause severe lacerations, their primary effect is to crush tissue, which causes death and necrosis of the skin and deeper planes, facilitating bacterial growth at the site. The jaw power of certain breeds of large domestic dogs can injure muscles, tendons, and bones (Figure 1)^6^ ^,^ ^7^ ^,^ ^8^ ^,^ ^9^.

Traumatic injuries caused by dog bites can lead to bacterial and fungal infections. Of the many bacteria present in canine oral flora, staphylococcal and streptococcal pathogens are common in human skin infections, but there are also less common agents such as gram-negative and anaerobic bacteria (*Eikenella corrodens, Pasteurella multocida, Capnocytophaga canimorsus, Proteus sp., Klebsiella sp., Haemophilus sp., Enterobacter sp.,* and *Bacteroides spp.*)^10^.

Domestic cat (*Felis catus*) bites differ clinically from dog bites in that their sharp teeth cause deep punctures rather than lacerations or crushing. Their saliva contains many bacteria, and there is a high chance of infection, which is further complicated by the difficulty of cleaning puncture wounds as opposed to laceration wounds^11^ ^,^ ^12^. Among the various infectious conditions that occur after cat bites, cat scratch disease (which also occurs after bites) caused by *Bartonella henselae* and infection by *Pasteurella multocida* can be severe and cause cardiac complications and sepsis. Infection can also be caused by other agents such as staphylococci, streptococci, *Actinomyces* sp., *Bacteroides* spp., *Fusobacterium* sp., *Clostridium* sp., *Propionibacterium* sp., *Fusobacterium* sp., *Wolinella* sp., *Porphyromonas* sp., *Prevotella* sp., and *Peptostreptococcus* sp^13^.

An infection that has become common in recent decades is sporotrichosis transmitted by domestic cats; it has taken on epidemic aspects in the state of Rio de Janeiro, Brazil, and is endemic throughout the country. The typical presentation of an inoculation ulcer and ascending lymphangitis facilitates its diagnosis; however, some atypical manifestations can delay disease identification and treatment^14^.

Other domestic animals can also cause serious injuries with copious bleeding and massive tissue loss. Bites from Equidae, such as horses (*Equus caballus*), ass (*Equus asinus*), and their sterile hybrids (donkeys and mules), are highly traumatic due to the force of their jaws. Injuries of this type are destructive, with large amounts of tissue and even fingers being ripped off. There are no records of infection from these bites because of their low frequency of occurrence and ability to reach emergency rooms. This injury profile may also occur rarely in cattle (*Bos taurus*) bites in rural areas^1^.

**FIGURE 2:**
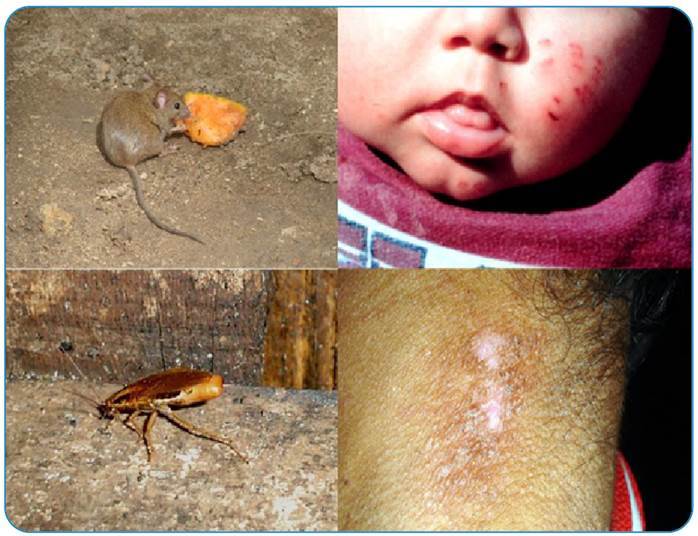
Above: rat bites on a child's face. Below: skin erosion caused by cockroach bites on an indigenous child. Both wounds have a high potential to become secondarily infected.

Invasive animals, including rodents (*Rattus rattus, Rattus norvegicus, Mus musculus*, black rats, brown rats, and mice) and cockroaches, are found in domestic environments. Rat bites are highly septic, putting victims at risk of serious infections and severe diseases such as rabies and tetanus. They occur in areas of low socioeconomic status, and children can be seriously injured if adequate parental care is not provided^1^. Wild rat bites can transmit *Streptobacillus moniliformis* and *Spirillum minus*, which can cause fever, myalgia, exanthematous eruptions, polyarthralgias, and lymphadenitis in humans; athough this is not the most common infection route, leptospirosis should be considered a complication of rat bites (Figure 2)^1^. Cockroaches (Blattidae Family) are insects that can erode human skin by feeding on keratin. As these animals move around in sewers and contaminated areas, lesions are likely to become infected in later stages (Figure 2)^15^.

## WILD ANIMALS

Monkey bites are relatively more common. This is because of the increasing presence of monkeys in urban areas or in green areas close to urban neighborhoods (parks), which causes the animals to approach humans in search of food or being raised as pets. Capuchin monkeys (*Sapajus* sp.) are the most common species associated with bites^16^.

**FIGURE 3:**
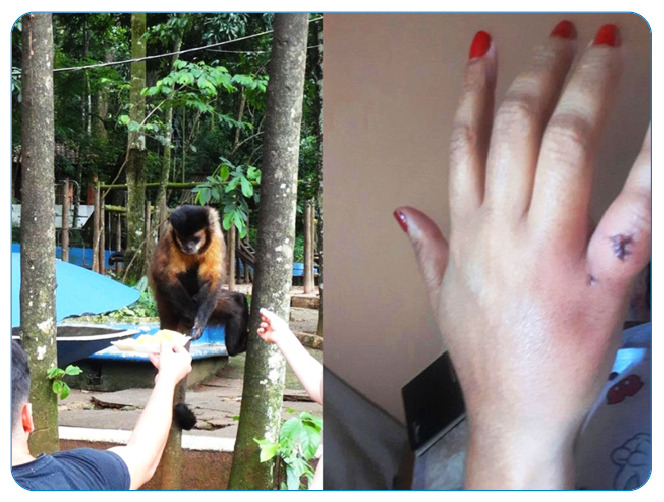
A capuchin monkey bite and late secondary infection on the hand of a woman who had the monkey as a pet. Bites can also occur when people approach and feed wild monkeys. Photos: Vidal Haddad Junior.

**FIGURE 4:**
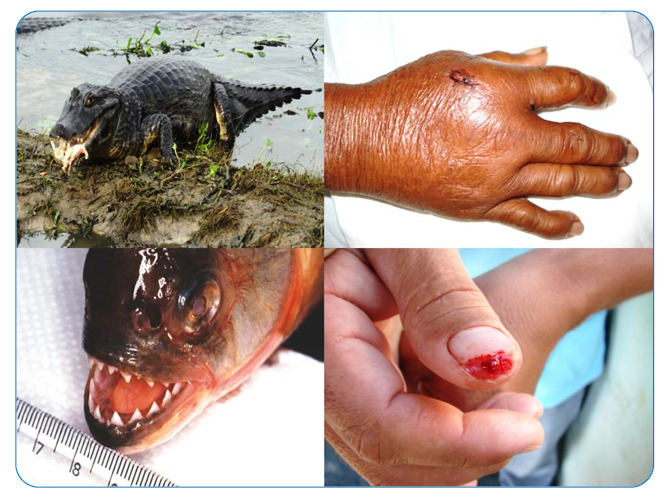
Above: a caiman bite showing severe secondary infection. Below: a piranha bite with tissue destruction of the thumb and part of the nail. These are bites with a high septic potential.

Their oral flora have recently been studied as they cause serious infections and are similar to human oral flora (Figure 3)^16^. Other uncommon injuries can be caused by jaguars (*Panthera onca*)^17^, tapirs (*Tapirus terrestris*)^18^, bats (including the vampires *Desmodus rotundus*), caimans^19^, snakes, tegu lizards, and fish, such as sharks, piranhas^20^, and trahiras or wolf fish (Figure 4). Bites from any of these animals are susceptible to bacterial infections, but special care needs to be taken in the prevention and control of infections by highly pathogenic agents, such as Aeromonas hydrophila and Vibrio vulnificus, which initially manifest as necrotizing fasciitis and sepsis, with high mortality rates^1^. Infections caused by staphylococci, *Proteus* sp., *Pseudomonas* sp., and *Clostridium tetani* are also common after venomous snake bites.

## BITES BY HUMANS

The composition of the human oral flora is complex, and it harbors germs capable of causing infections transmitted by saliva via bites. The conditions under which these bites occur in other humans are fights and prisons. Human oral flora is similar to that of other primates.

**FIGURE 5:**
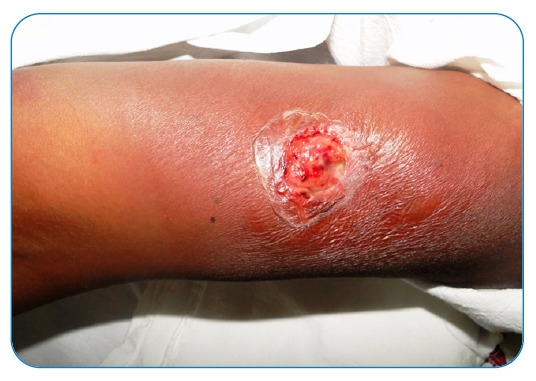
Ulcers caused by human bites are highly likely to become infected by the complex flora of the oral cavity. In this case, cellulitis appeared after the bite.

A human bite can tear the victim’s tissue or cause major lacerations. They mainly occur on the hands, an area where infections are a serious problem due to the difficulty of cleaning, the presence of terminal circulation, and the large number of delicate and specific tendons, vessels, and muscles. Scalp lesions are also common (Figure 5). In a previous study, the bacterial agents in human bite injuries were found to be similar to those isolated from mammalian bites (staphylococci, streptococci, *Eikinella corrodens, Haemophilus influenzae, Fusobacterium nucleatum, Peptococcus* sp., *Peptostreptococcus* sp., *Porphyromonas* sp., and *Prevotella* sp.)^5^. A 1987 study of almost 450 human patients with bite injuries showed an approximately 20% bite infection rate after human bites in almost all patients, which should be considered when treating this type of aggression. However, this is not indicative of the use of antimicrobial prophylaxis or surgery for debridement because intensive cleaning of an open wound can prevent infections^5^. There have also been reports of human immunodeficiency virus transmission following a human bite^21^. The most common infections are caused by staphylococci, streptococci, fusospiral bacteria, and *Eikenella corrodens* ^5^.

## TREATMENT

Infections after animal bites are rarely caused by a single agent, with the majority having a mixed etiology. *Pasteurella multocida* infection deserves further attention. This gram-negative bacterium can be found in the saliva of both domestic and wild dogs and cats, causing infections of varying severities. The most severe bite injuries can progress to rapid septicemia^22^ ^,^ ^23^ ^,^ ^24^. *Capnocytophaga canimorsus* is a gram-negative, rod-shaped bacterium that can cause disseminated intravascular coagulation and rapid sepsis^24^.

Animal bites are generally located on the extremities, but they can also be observed in a victim's cephalic segment. Hand infections have a high risk of developing complications and permanent motor sequelae. Careful wound cleaning is important for preventing infections, especially in laceration and puncture wounds^22^ ^,^ ^23^ ^,^ ^24^. Animal bites should be treated according to their initial manifestations (bleeding and tissue loss) and subsequent complications, such as infections, extensive necrosis, and esthetic repair of large lesions^25^ ^,^ ^26^.

The patient must be reassured at the time of injury, during emergency care, and during transportation, and the health team must wear gloves for mutual protection during care. Bleeding can be stopped by compressing the wound with clean compresses. If bleeding does not put the patient at risk, the most important measure is intense and thorough asepsis of the wound using soap and water. If pressurized water jets can be used, the results will be more effective^25^.

A good measure is to use a saline syringe and internally wash puncture wounds, such as those caused by felines. Achieving asepsis is the most important aspect of this type of injury, which must always be considered by the medical team^26^. Devitalized tissues at the edges of wounds (for example, in crushing dog bites) need to be removed, and it is important to reassess the area in a day or two if there is any doubt regarding tissue vitality. Sutures should be avoided because of the risk of infection; however, spaced suturing is possible when bleeding is uncontrollable. This is not valid for wounds in problem areas, such as hands, or puncture wounds^26^. In later stages, secondary suturing may be performed if there are no infections.

Local inflammatory signs (erythema, edema, and purulent discharge) and fever in the late stages may indicate infection. It is essential to perform a bacterial culture test and antibiogram in the early stages because of the great diversity of agents. Antibiotics are essential, and the first choice (even before culture and antibiogram results) is an amoxycillin/clavulanic acid combination (500 mg every 8 h or 875 mg every 12 h for 10 days). This option is valid for all animal bites, including reptiles and fish^1^ ^,^ ^25^ ^,^ ^26^ Another good option is cephalexin 500 mg every 6 h for 10 days^1^ ^,^ ^25^ ^,^ ^26^. Patients without tetanus immunization should receive tetanus toxoid (the vaccine) and, in some cases, immunoglobulin against the tetanus toxin^25^.

Bites from wild mammals (and domestic animals of unknown origin) carry the risk of infection with the rabies virus and the *Clostridium tetani* bacterium. Rabies must be prevented in wild mammal bites. Little is known about the transmission of infection by frugivorous and insectivorous bats. Any injury caused by an unknown wild or domestic animal requires prophylaxis, a course of five intramuscular vaccine doses (on days 0, 3, 7, 14, and 28), and, in severe cases, rabies immunoglobulin serum (20 IU/kg) infiltrated into the bite site^27^ ^,^ ^28^.

## CONCLUSIONS

Despite the lack of data on this problem, animal bites are not uncommon in emergency rooms. Wild animals rarely attack, and most attacks occur when they are provoked or cornered. This is the reason why hands and feet are the main injury sites in humans, known as defence injuries. Wild animal bites are generally caused by animals kept as pets in domestic environments, but they can also occur in natural environments, injuring tourists and professionals, such as biologists, veterinarians, animal handlers, and hunters. However, domestic animals show another pattern of aggression, especially dogs trained to be ferocious, which causes serious and disfiguring injuries and copious bleeding. Contact with wild animals should always be avoided, no matter how harmless it may seem, due to the risk of traumatic injuries and subsequent complications. Domestic animals pose less risk, but we must remember that some diseases transmitted by them come from minimal injuries, and care must always be taken, especially with children. These simple measures prevent most accidents and their infectious complications. Health teams in emergency care centers should be aware of the initial and late treatment measures needed to control this problem.
